# Selective non-enzymatic electrochemical detection of dopamine using nickel molybdate nano-dots anchored on CNT fiber microelectrodes

**DOI:** 10.1039/d5ra05187h

**Published:** 2025-10-02

**Authors:** Hamza Arif, Arfaa Sajid, Abid Ali, Nimra Ahmed, Munawar Iqbal, Salih Akyürekli, Murat Kaleli, Norah Alwadai, Umer Shafique, Arif Nazir

**Affiliations:** a Department of Chemistry, The University of Lahore Lahore Pakistan anmalik77@gmail.com; b School of Chemistry, University of the Punjab Lahore Pakistan; c Innovative Technologies Application and Research Center, Suleyman Demirel University 32260 West Campus Çünür Isparta Turkey; d Department of Physics, College of Sciences, Princess Nourah bint Abdulrahman University P.O. Box 84428 Riyadh 11671 Saudi Arabia; e Department of Chemistry, Government College University Lahore Pakistan

## Abstract

This research demonstrates the hydrothermal synthesis of α-nickel molybdate nano-dots (α-NiMoO_4_) and their fabrication on a carbon nanotube fiber (CNTF), serving as an electrocatalyst for non-enzymatic electrochemical sensing of dopamine (DA). Several analytical techniques, such as, scanning electron microscopy (SEM), X-ray diffraction (XRD), energy-dispersive X-ray spectroscopy (EDX), and Fourier transform infrared spectroscopy (FTIR), were employed for morphological analysis, crystallinity and elemental composition analysis and characteristic bond vibration analysis of NiMoO_4_, respectively. The characterization revealed the presence of ∼10 nm α-NiMoO_4_ nano-dots with a significant amount of NiO. The NiMoO_4_@CNTF electrode exhibited a remarkable sensitivity and selectivity towards DA, despite the presence of other interfering species, including glucose (Glu), uric acid (UA), ascorbic acid (AA), urea and NaCl. A comparative study of the bare CNTF and NiMoO_4_@CNTF electrode revealed the boosted capabilities of the fabricated flexible microelectrode with a sensitivity of 2.02 mA cm^−2^ mM^−1^, a detection limit of 58.2 μM, and a quantitation limit of 0.176 mM, with a linearity range of 0.1–1.2 mM. In comparison, bare CNTF exhibited a sensitivity of 1.4 mA cm^−2^ mM^−1^, a detection limit of 107 μM, and a quantitation limit of 0.321 mM, with a linearity range of 0.1–0.7 mM. The enhanced performance of NiMoO_4_@CNTF was attributed to an increase in the active sites due to the large surface area of the NiMoO_4_ nano-dots, causing fast electron kinetics and improving the sensor's sensitivity and reliability. The outcomes of this study could foster innovation in designing electrochemical sensors based on metal molybdates/double metal oxides with carbon-based nanomaterials for sensing electroactive biomolecules through real-time medical analysis.

## Abstract 2

NiMoO_4_ nano-dots anchored on CNTF microelectrodes for the selective, non-enzymatic electrochemical detection of dopamine, showing high sensitivity, low detection limit, and excellent selectivity against interfering biomolecules.

## Graphical abstract

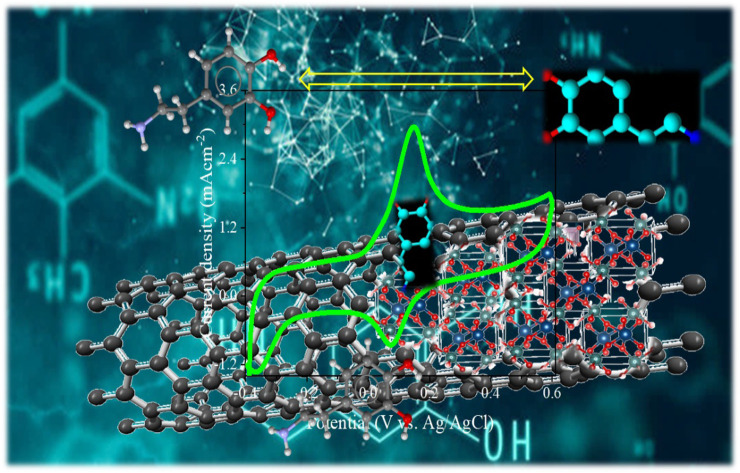

## 1. Introduction

Dopamine (DA) is one of the many important neurotransmitters, and many biological processes in the human body, such as memory, mood, learning, emotional responses, and sleep, are related to DA levels. Abnormal variation in DA levels in the human body can lead to neurodegenerative diseases such as Parkinson's disease, Alzheimer's disease and schizophrenia.^1,2^ It can also contribute to conditions like attention deficit hyperactivity disorder (ADHD) in children.^3^ Early diagnosis plays a crucial role in treating such medical conditions. Electrochemical sensors allow real-time monitoring and provide accurate measurements and are considered portable, fast and inexpensive. These characteristics can help in medical therapies that require frequent monitoring of DA levels in the patient's body to maintain or restore optimal levels of DA.^4,5^ Therefore, a rapid detection system for DA levels in the human body is necessary for effective treatment. The conventional techniques for DA detection include high performance liquid chromatography,^6^ enzyme-linked immunosorbent assay, electrophoresis,^7^ spectroscopy,^8^ and chemiluminescence.^9^ Such techniques are highly sensitive and accurate, but they are not easy to handle, require expensive instruments and skillful operators, and are time-consuming and lab-based; hence, they are not suitable for frequent DA monitoring. Electrochemical sensors can bypass all these drawbacks, especially when integrated into a portable DA sensing device.^10^

Enzymes and aptamers are highly specific and sensitive for sensing applications, but they degrade easily under physiological conditions, causing long-term reliability issues, limited reusability and low reproducibility.^11^ In electrochemical sensing, pristine electrodes face problems as their oxidation peak potentials overlap with each other.^12^ To solve this problem, researchers are looking at advanced nanomaterials to achieve a significant improvement in sensor performance.^13^ Integration of advanced nanomaterials into electrochemical sensors can achieve high sensitivity, specificity, low limits of detection, cost effectiveness and long-term stability.^14^

Lately, different materials such as carbon nanotube fibers, graphene and its derivatives, quantum dots, metal oxides, double metal oxides,^15^ polymers and metal–organic frameworks have shown great results in the detection of biomolecules.^16^ A sensor with LaV-MWCNTs modification has shown good sensitivity with a detection limit (DL) of 0.04 μM for DA.^17^ Similarly, an electrode with EuO_3_@Cr_2_O_3_ modification has shown a sensitivity of 0.0013 mA cm^−2^ mM^−1^ and a DL of 0.7 μM.^18^ However, these modified electrodes have limited accuracy and real-time efficiency because of their poor electrocatalytic activity and electrical conductivity.^19^

The double metal oxides have shown outstanding electrochemical activity. The catalytic activity of double metal oxides in comparison with metal oxides is significantly higher.^20^ Double metal oxides exhibit great thermal and chemical stability, and good electronic conductivity, electro-catalytic properties and electro-optical effects. These characteristics make them a good choice for sensing applications, water splitting, supercapacitors, lithium-ion batteries and as photocatalysts.^21^ Double metal oxides significantly enhance the properties of electrochemical sensors by improving their sensitivity, specificity/selectivity, accuracy, limit of detection and reproducibility.^22^

In recent years, metal molybdates have been a center of attention due to their high electrical conductivity and feasible oxidation state. Nickel molybdate (NiMoO_4_) belongs to the family of metal molybdates and has gained a lot of attention because of its chemical stability, enhanced electrochemical activity and cost effectiveness. Electrodes modified with NiMoO_4_ have shown remarkable results; *i.e.* low limits of detection, wide linear range, anti-interference properties, high sensitivity and cost-effectiveness.^23^

This research demonstrates a comparative study of a bare CNT fiber-based electrode and an NiMoO_4_-modified CNT fiber-based electrode for the detection of DA by means of electrochemical experiments, such as cyclic voltammetry, chronoamperometry and interference studies. Various sophisticated techniques, including SEM, EDX, XRD and FTIR, were employed for the characterization of NiMoO_4_. This approach gives insight into the enhanced electrocatalytic activity and anti-interferent features brought by NiMoO_4_ for the detection of DA. Most well-known interferents of DA were examined; *i.e.* glucose, urea, NaCl, ascorbic acid (vitamin C), and uric acid. The study takes a hybrid approach by combining materials chemistry with electrochemistry to achieve a specific DA detection in the presence of interferents. The resulting outcome has possible implications in neurochemical investigations, portable sensors and real-time clinical diagnostics. NiMoO_4_@CNTF demonstrates the potential to overcome interference from other electroactive species in biological fluids, making it highly specific for DA detection.

## 2. Materials and methods

### 2.1 Materials

In this study, all chemical reagents and solvents were of analytical grade and employed without additional purification. The following reagents were procured from Merck: nickel chloride hexahydrate (NiCl_2_·6H_2_O, 99.9%), sodium molybdate dihydrate (Na_2_MoO_4_·2H_2_O, 99.9%), ethanol (C_2_H_6_O, 99.8%), sulfuric acid (H_2_SO_4_, 37%), dopamine (C_8_H_11_NO_2_, 99.9%), Nafion solution, ascorbic acid (C_6_H_8_O_6_, 99.8%), glucose (C_6_H_12_O_6_), urea (CH_4_N_2_O, 99.9%), uric acid (C_5_H_4_N_4_O_3_, 99.9%), sodium chloride (NaCl, 99.9%), monopotassium phosphate (KH_2_PO_4_, 99.9%), and dipotassium phosphate (K_2_HPO_4_, 99.9%). Throughout the experiment, laboratory-grade double-distilled water was utilized.

### 2.2 Synthesis of NiMoO_4_ nano-dots

The NiMoO_4_ nano-dots were synthesized hydrothermally by following a previously established procedure.^24^ NiCl_2_·6H_2_O (0.0075 moles, 1.78 g) and Na_2_MoO_4_·2H_2_O (1.81 g) were weighed and mixed by adding 50 mL of double-distilled H_2_O under magnetic stirring for 40 minutes at 25 °C. The obtained homogenized solution was transferred to a 150 mL Teflon-lined autoclave, capped tightly, and kept at 150 °C for 8 hours in an electronic oven. The autoclave was then allowed to cool down to room temperature, and the obtained solution was centrifuged to remove excess water. The obtained product was washed several times with double-distilled H_2_O, then dried at 60 °C in an electronic oven overnight. The product was then calcinated at 400 °C for 2 hours and allowed to cool down to room temperature.

### 2.3 Electrode fabrication

In order to fabricate the CNT fiber with NiMoO_4_, a functionalization procedure was first performed by dipping a CNT fiber (2 cm in length) in conc. H_2_SO_4_ for 10 minutes. Afterwards, a cleaning process was performed using distilled water and ethanol *via* rapid ultrasonication. The functionalized CNT fiber was then dried in an oven at 40 °C for 2 hours. NiMoO_4_ nano-dots (10 mg) were dispersed in a solution of Nafion polymer (2 mL, 0.5 wt%) with (water: ethanol) by subjecting the mixture to ultrasonication for 30 minutes to ensure uniform dispersion. Then, 5 μL of the prepared NiMoO_4_ nano-dot slurry was drop-cast on the functionalized CNT fiber and dried in an electronic oven at 40 °C for 1 hour. The prepared CNTF was then attached to a glass plate (2.5 cm × 1 cm) with Teflon tape at one end and indium metal soldered on the other end. The prepared electrode plate was then screwed onto an electrode holder, where the soldered indium on the electrode plate came in contact with the electrode holder's screw.

### 2.4 Characterizations

The morphological features were investigated through scanning electron microscopy (SEM) (FEI Quanta FEG 250). Analysis of the crystalline structure and phase composition was performed *via* X-ray diffraction (XRD) (Bruker D8 Advanced TWIN-TWIN). Fourier transform infrared spectroscopy (Bruker) was carried out to study the chemical composition and functional groups. Elemental composition was analyzed *via* energy-dispersive X-ray spectroscopy (EDX). All electrochemical measurements were conducted in a three-electrode system, in which the modified CNT fiber was employed as the working electrode, with Ag/AgCl as the reference electrode and platinum wire as the auxiliary electrode. A 0.1 M potassium phosphate (KH_2_PO_4_ and K_2_HPO_4_, with pH 8) solution was used as the supporting electrolyte for all electrochemical measurements. Cyclic voltammetry, staircase chronoamperometry, and amperometric interference analysis were carried out for the electrochemical analysis. All electrochemical measurements were carried out at room temperature.

## 3. Results and discussion

### 3.1 SEM and EDX

**Fig. 1:**
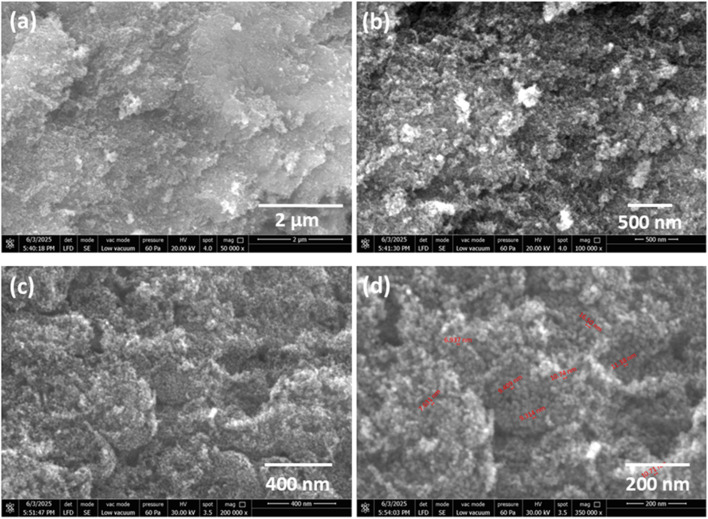
SEM images of NiMoO_4_ nano-dots at (a) 2 μm, (b) 500 nm, (c) 400 nm, and (d) 200 nm.

Scanning electron microscopy is primarily used to investigate the surface topography, morphology and compositional characteristics of various materials. SEM analysis at different resolutions gives insight into the surface morphology of NiMoO_4_ nano-dots, which appear to be highly textured^23,25^ with an average size of 10 nm. The SEM images presented in Fig. 1(a)–(d) illustrate the surface morphology of NiMoO_4_ at different resolutions of 2 μm, 500 nm, 400 nm and 200 nm, respectively. Fig. 1(a) gives insight into the overall surface texture and morphology. At higher resolution, the sphere-like nano-dots can be seen, as shown in Fig. 1(d). SEM analysis revealed that NiMoO_4_ nano-dots possess a high surface area due to their smaller particle size distribution, making the material a highly efficient electrocatalyst.

SEM post-analysis of the electrocatalyst (NiMoO_4_) surface offers important information on the structural alterations and morphological stability following electrochemical reactions, as given in the SI (Fig. S1). Particle aggregation, surface roughness, porosity retention, and potential deterioration or detachment of active sites that may occur during electrocatalysis are all assessed and observed to be well maintained after the electrocatalytic sensing process.

**Fig. 2:**
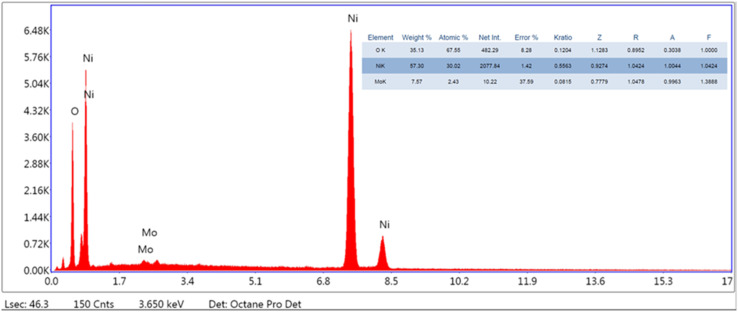
EDX analysis for assessing the elemental abundance of the synthesized material.

EDX analysis was also employed to study the elemental composition of the synthesized material. The EDX spectrum shown in Fig. 2 reveals clear peaks of nickel (Ni) and molybdenum (Mo) along with a peak of oxygen (O), which validates the synthesis of the NiMoO_4_ nano-dots. Ni shows stronger characteristic X-ray lines, which are efficiently excited and detected by EDX, while Mo shows peaks lower in energy and weaker in intensity due to lower X-ray generation efficiency, or higher absorption within the sample or detector window, or due to overlapping signal with noise. Subsequently, if nickel is more enriched at the surface or exists as unreacted Ni species, it may dominate the EDX signal; consequently, Mo may be present in a more dispersed or poorly crystalline form or may not be able to fully react with Ni, causing inhomogeneity in the composition. In hydrothermal synthesis, excess Ni^2+^ could possibly precipitate preferentially, causing Ni-rich surface layers. Overall, the synthesized material showed high purity because only relevant peaks appeared in the spectrum.^26^ The EDX analysis confirms the successful synthesis of NiMoO_4_ nano-dots.

### 3.2 Elemental mapping

**Fig. 3:**
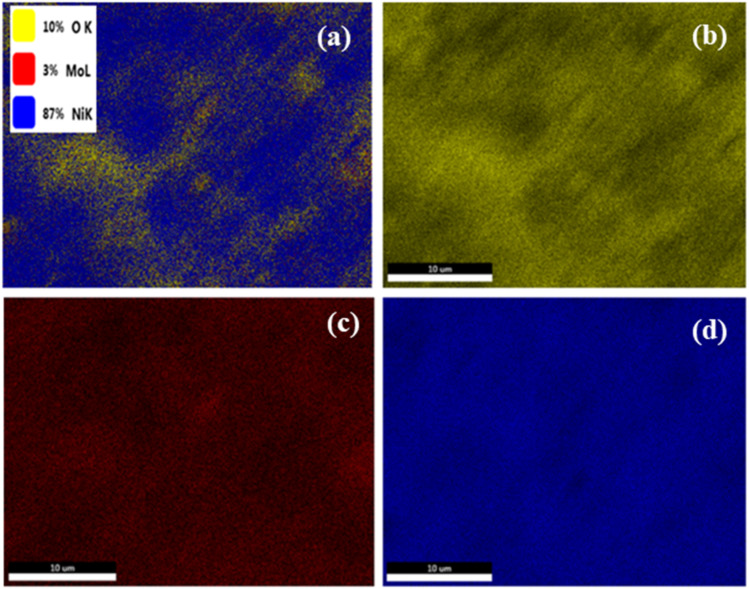
EDX elemental mapping for NiMoO_4_ nano-dots: (a) survey analysis, (b) oxygen, (c) molybdenum and (d) nickel.

Analysis of the elemental mapping images confirms the presence of nickel (Ni), molybdenum (Mo) and oxygen (O) in the synthesized NiMoO_4_ nano-dots, as shown in Fig. 3. The mapping reveals that all three elements are evenly dispersed throughout the scanned region, indicating the formation of a homogeneous composite without any aggregation. The oxygen distribution overlaps uniformly with those of Ni and Mo, confirming the oxide nature of the material.

### 3.3 XRD and FTIR

**Fig. 4:**
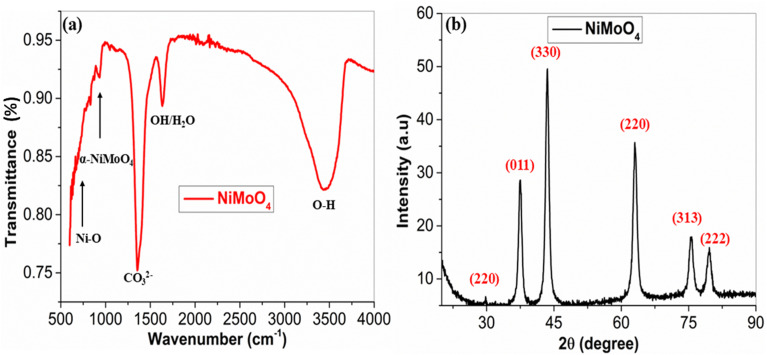
(a) FTIR analysis of NiMoO_4_ and (b) XRD analysis of NiMoO_4_.

The crystallographic and structural features of the NiMoO_4_ nano-dots were studied by X-ray diffraction, as shown in Fig. 4(b). α-NiMoO_4_ exhibits a signature peak (220) at ≈28.8°^27^ and another reflection of α-NiMoO_4_ (330) at 43.9°.^28^ Other intense peaks point towards NiO planes (200), (220), (311), (222) at 43.3°, 62.9°, 75.5°, 79.4°, respectively.^29^ The sample is predominantly NiO, with minor amounts of α-NiMoO_4_ (monoclinic)phase, while other β-NiMoO_4_ and NiMoO_4_·H_2_O phases are absent. The peak at ≈44° overlaps α-NiMoO_4_ (330) and NiO (200). The peak at ≈38° is likely an impurity, most probably Ni(OH)_2_ from residual nickel in the hydrothermal reaction. In the absence of a strong acid or mineralizer, nickel ions can hydrolyze under hot aqueous conditions, especially when basic molybdate is present, to form a Ni(OH)_2_ precipitate. Most Ni(OH)_2_ converts into NiO upon annealing at 400 °C, but a minor fraction can remain or transiently reform upon cooling to give a distinct Ni(OH)_2_ peak. Hence, the peak (011) at 38.2° is possibly due to the presence of Ni(OH)_2_. Standard XRD references (JCPDS cards 33-0948 for α-NiMoO_4_, 47-1049 for NiO and 14-0117 for Ni(OH)_2_) were used. The XRD pattern demonstrates features from NiO, α-NiMoO_4_ and Ni(OH)_2_.

A broad O–H stretching band can be seen at ∼3500 cm^−1^, which is a characteristic of hydrogen-bonded OH groups or adsorbed water.^30^ A sharp peak ∼1350–1400 cm^−1^ indicates an impurity, *i.e.* residual carbonate or nitrate, because surface CO_3_^2−^ (carbonate) from CO_2_ absorption can absorb near ∼1360–1380 cm^−1^. In the absence of organic additives (eliminates C–H or C=H), a band like this is usually due to inorganic residues, *e.g.* CO_3_^2−^, from precursors or atmospheric CO_2_. The fingerprint vibrations of NiMoO_4_ can be seen at ∼750–1000 cm^−1^, NiMoO_4_ contains MoO_4_ units and Ni–O bonds. Mo–O stretching modes from MoO_6_ octahedra in α-NiMoO_4_ or MoO_4_ units in some polymorphs usually appear at 800–950 cm^−1^, which can be seen clearly in Fig. 4(a). The α-NiMoO_4_ (monoclinic) phase exhibits strong Mo–O–Mo and Ni–O–Mo stretches around 946 and 858 cm^−1^. In α-NiMoO_4_, peaks around 858–946 cm^−1^ (Mo–O–Mo and Ni–O–Mo stretches) are expected. Ni–O stretches and bending modes usually lie below 700 cm^−1^ at around 586 and 414 cm^−1^. The observed lattice matches the α-NiMoO_4_ (monoclinic) phase. The peaks at ∼1600 cm^−1^ and ∼3500 cm^−1^ are due to surface OH/water.

### 3.4 Detection of DA *via* electrocatalytic activity

**Fig. 5:**
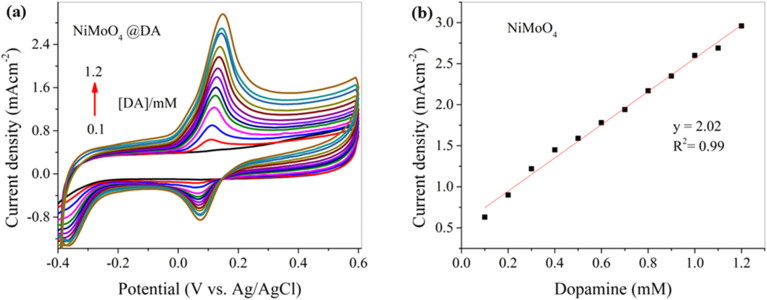
(a) CV curve of the NiMoO_4_@CNTF electrode for different concentrations of DA at a fixed scan rate of 0.10 V s^−1^ in 0.1 M (KH_2_PO_4_, K_2_HPO_4_, pH 8) and (b) its calibration curve.

Cyclic voltammetry and chronoamperometry were employed for the electrochemical detection of DA *via* the NiMoO_4_@CNTF modified electrode. A distinct anodic peak appears for DA at 0.12 V *vs.* Ag/AgCl, as shown in Fig. 5(a). The oxidation of DA at the anode yields dopamine-*o*-quinone, which is the initial and primary oxidation product of DA. During oxidation, DA loses two electrons and two protons to form dopamine-*o*-quinone. The shape of the peak indicates fast electron transfer kinetics on the electrocatalytically active surface of NiMoO_4_, which also means that the redox reaction is electrochemically reversible. The appearance of the cathodic peak at 0.08 V shows the reduction process in which the electrode allows dopamine-*o*-quinone to reduce back to dopamine.^2^ The smaller reverse cathodic peak points towards quasi-reversible kinetics. The electrode sensitivity over a DA concentration range of 0.1 to 1.2 mM demonstrates a linear relation, as shown by the calibration curve in Fig. 5(b), with a corresponding *R*^2^ value of 0.99. According to the slope in Fig. 5(b), by increasing DA concentration, higher anodic currents are observed due to the rules of diffusion-controlled electron transfer.

**Fig. 6:**
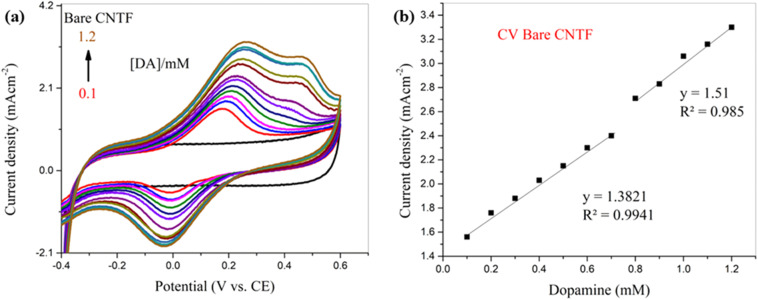
(a) CV curve of the bare CNT fiber electrode with different concentrations of DA at a fixed scan rate of 0.10 V s^−1^ in 0.1 M (KH_2_PO_4_, K_2_HPO_4_, pH 8) and (b) its calibration curve.

For a comparative analysis, a bare CNT fiber electrode was used for the detection of DA before modification under the same conditions to evaluate the true effect of the NiMoO_4_ for enhancing the electrochemical properties of the working electrode. The cyclic voltammograms recorded with the bare CNT fiber electrode for DA detection and its calibration curve are shown in Fig. 6(a) and (b), respectively.

The cyclic voltammogram shown in Fig. 6(a) reveals the electrochemical behavior of the bare CNT fiber towards DA detection, which demonstrates poorly shaped, broad peaks and also follows quasi-reversible kinetics. The calibration curve shown in Fig. 6(b) shows two distinct slopes, revealing a fairly linear relation between current density and DA concentration with *R*^2^ values of 0.99 and 0.98, respectively. The NiMoO_4_-modified CNT fiber showed significantly enhanced electrochemical behavior, with reduced overpotential, high sensitivity, sharp peaks and improved electron transfer kinetics due to its larger surface area, superior conductivity, and abundance of active sites. Although the current density with the bare CNT fiber electrode was higher when compared to that of the NiMoO_4_-modified CNT fiber electrode, the NiMoO_4_@CNTF electrode offered approximately 44% higher sensitivity than the bare CNT fiber electrode.

**Fig. 7:**
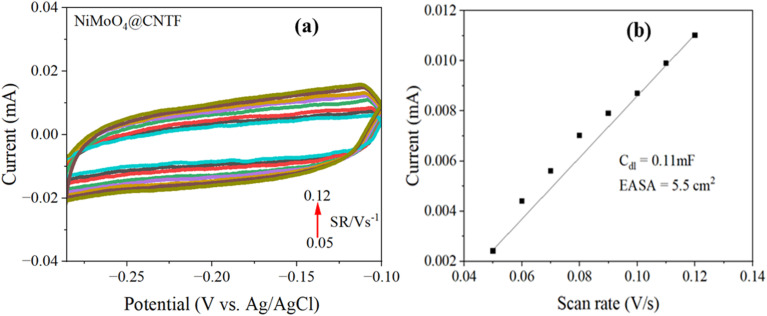
(a) Non-faradaic CV curves of NiMoO_4_@CNTF at increasing scan rates and (b) corresponding plot of capacitive current density *versus* scan rate.

To quantify the electrochemically accessible interface, we measured cyclic voltammograms (Fig. 7(a)) in the non-faradaic potential window and extracted the double-layer capacitance (*C*_dl_) from the slope of capacitive current *versus* scan rate. The NiMoO_4_@CNTF yields *C*_dl_ = 0.11 mF. Using the specific capacitance value of (*C*_s_ 0.02 mF cm^−2^), this corresponds to an electrochemically active surface area (EASA) *C*_dl_/*C*_s_ = 5.5 cm^2^. The calculated EASA in Fig. 7(b) is substantially larger than the geometric exposure of the CNT fiber (1 cm^2^), indicating that the NiMoO_4_@CNTF nano-dot integration multiplies the real active surface area.

### 3.5 Effect of scan rates

**Fig. 8:**
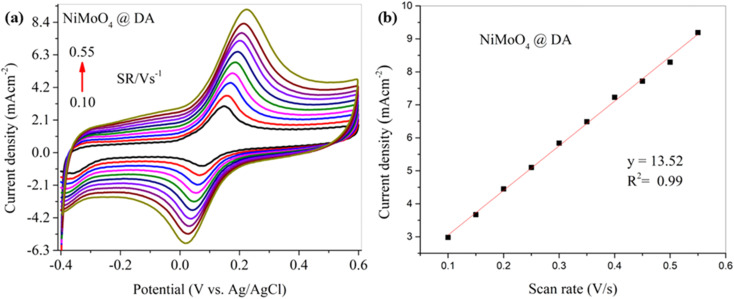
(a) CV curve for DA detection on the NiMoO_4_@CNTF modified electrode at various scan rates (0.10 to 0.55 V s^−1^) and (b) corresponding calibration curve of current density *vs.* scan rate.

The reaction dynamics for DA detection were determined through various scan rates ranging from 0.10 to 0.55 V s^−1^. Fig. 8(a) illustrates a cyclic voltammogram for DA detection based on the NiMoO_4_@CNTF electrode at different scan rates. As the scan rate gradually increases, a parallel increase of both anodic and cathodic peak current densities can be observed due to enhanced mass transport. The shape of the voltammogram is fairly symmetrical, and it illustrates the stability of the electrochemical process taking place, indicating that the process is electrochemically reversible even at higher scan rates, where a shift in the peak position can sometimes be seen due to reduced reversibility as adsorption effects take place, and the process becomes more kinetically controlled than diffusion-controlled. The cyclic voltammogram shown in Fig. 8(a) demonstrates fast reaction kinetics for DA detection. In Fig. 8(b), the calibration curve illustrates a proportional response of current density with the scan rate.

In Fig. 8(b), the *R*^2^ value of 0.99 demonstrates a strong linear correlation between the scan rate and the response of the modified electrode. The linear pattern of response from the modified working electrode for DA detection at various scan rates illustrates the excellent reliability. The linear correlation indicates that the electron transfer happens predominantly at the electrode interface rather than depending on DA diffusion through the solution, which demonstrates a surface-controlled behavior of the reaction. The NiMoO_4_@CNTF electrode thus proves to be effective and versatile in its performance for DA detection. Conclusively, the electrode shows a steady electrochemical response at different scan rates for DA detection, with a strong linear correlation recorded in the calibration curve.

**Fig. 9:**
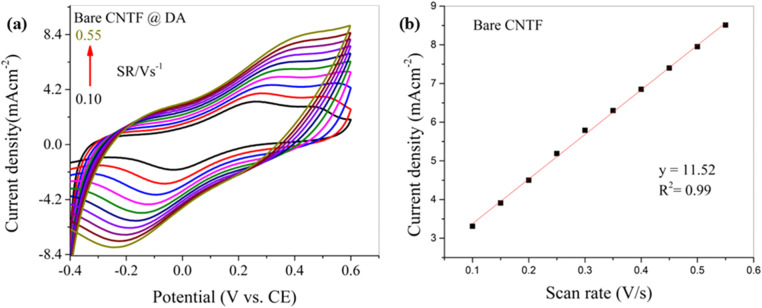
(a) CV curve for DA detection on the bare CNTF electrode at various scan rates (0.10 to 0.55 V s^−1^) and (b) corresponding calibration curve of current density *vs.* scan rate.

Similarly, the bare CNT fiber electrode was analyzed to measure its response at various scan rates under the same conditions used for the modified electrode. The obtained cyclic voltammogram is shown in Fig. 9(a). A parallel increasing response in peak current densities (both anodic and cathodic) can be observed as the scan rate increases. The obtained voltammogram indicates a steady behavior (*R*^2^ = 0.99), but the drastic shift in peak positions with increasing scan rate illustrates poor reversibility and the reaction is considerably more kinetically controlled than diffusion controlled. This indicates that either the adsorption of the analyte on the electrode surface occurs or the electrode exhibits slow electron kinetics, making the electron transfer more one-sided or difficult to reverse. This poor reversibility in the CV indicates hindered electrochemical behavior. This type of adsorption can lead to surface fouling on the electrode by blocking the active sites or forming a residual film, preventing further reactions. For a sensor to be reusable, the electrode surface must return to its original state after each measurement; if the adsorbed species cannot desorb easily, the electrode cannot fully recover, making each new scan less accurate. As a result, the sensor loses its efficiency. Fig. 9(b) shows the calibration curve of the bare CNT fiber electrode for DA detection at various scan rates.

### 3.6 Square wave voltammetry

**Fig. 10:**
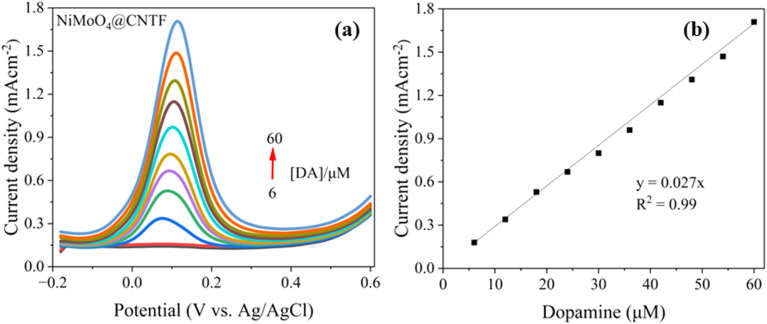
(a) SWV curve of the NiMoO_4_@CNTF electrode for different concentrations of DA in 0.1 M (KH_2_PO_4_, K_2_HPO_4_, pH 8) and (b) its calibration curve.

Dopamine detection with the NiMoO_4_@CNTF modified electrode was further examined by square wave voltammetry (SWV), a technique that enhances signal clarity by reducing capacitive interference. As shown in Fig. 10(a), a distinct anodic peak appeared at 0.011 V *vs.* Ag/AgCl, and its current increased steadily with dopamine concentration ranging from 6 to 60 μM. The peak potential remains almost unchanged across the concentration range, indicating stable and efficient electron transfer kinetics at the electrode surface. The corresponding calibration plot in Fig. 10(b) displays an excellent linear relationship (*R*^2^ = 0.99) with a slope of 0.027 mA cm^−2^ Mm^−1^ evidencing high analytical sensitivity.

### 3.7 Staircase chronoamperometry analysis

**Fig. 11:**
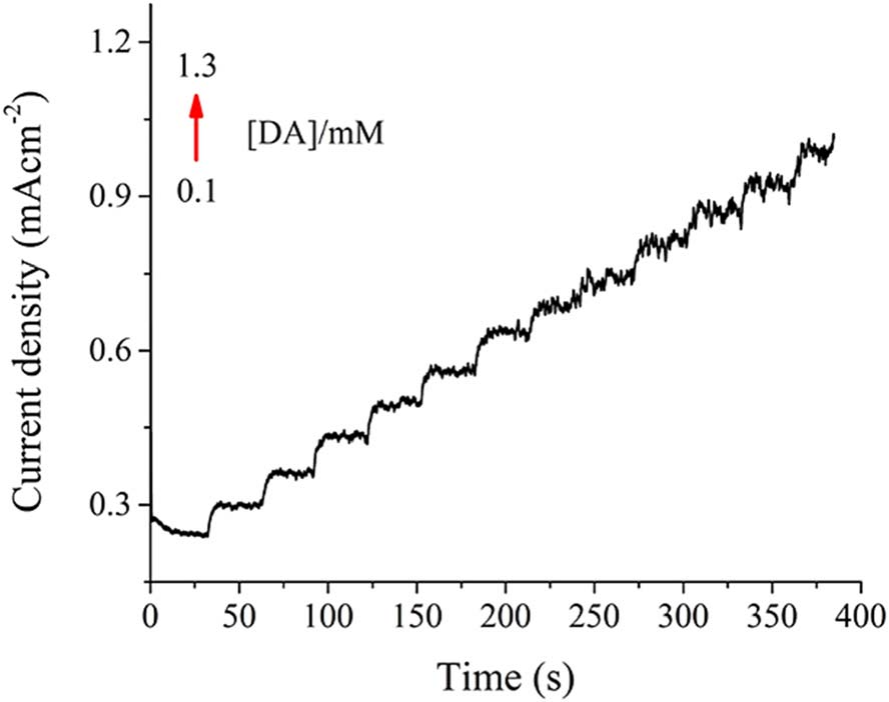
Staircase chronoamperometry response of the NiMoO_4_@CNTF electrode to DA at various concentrations in the range of 0.1–1.3 mM.

In order to test the electrochemical sensing ability of the NiMoO_4_@CNTF electrode for DA detection, staircase chronoamperometry was employed. For DA detection, the NiMoO_4_@CNTF electrode illustrated its response as stair-like steps, where the *x*-axis represented time (s) and the *y*-axis represented current density (mA cm^−2^), as demonstrated in Fig. 11. The sensor exhibits an increase in current density, ranging from 0 to 1.2 mA cm^−2^, over the time of 400 s for a DA concentration range of 0.1–1.3 mM. The linear correlation shown in Fig. 8 represents high sensitivity towards minor variations in solution concentration while offering a wide detection range and avoiding current saturation.

The distortion at higher analyte concentrations in the last few steps of the staircase could be due to residual capacitive (charging) currents. At each step, there is a fast-decaying capacitive current; at higher concentrations of analyte (DA) or with a short step duration, the end step residual current is not fully settled and contributes to the measured current, causing a distortion in the baseline. As the current increases with higher analyte concentrations, the solution resistance becomes more significant and the ions near the electrode's surface are consumed faster than they can be replaced, which causes polarization and creates distortions, especially later in the experiment.

### 3.8 Detection and quantitation limits of NiMoO_4_@CNTF

Detection and quantitation limits were calculated by using the cyclic voltammogram and square wave voltammogram's calibration curve shown in Fig. 5(b) and 10(b), its standard deviation (*σ*), and slope, as given in eqn (1) and (2).1
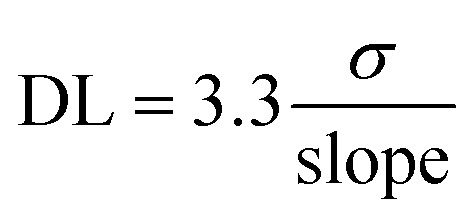
2
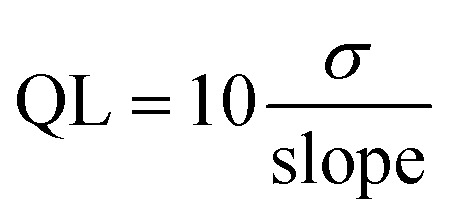


The detection limit (DL) (minimum amount of analyte that can be detected reliably) of 2.2 μM, quantitation limit (QL) (minimum amount of analyte required for reliable quantitative measurements) of 7.4 μM, and a sensitivity of 0.027 mA cm^−2^ mM^−1^ were measured with NiMoO_4_@CNTF, with a linearity range of 6–60 μM. This data demonstrates the ability of the sensor to detect and measure DA accurately and precisely. For comparison, the bare CNTF electrode demonstrated a higher DL and a sensitivity with a narrow linearity range. Cyclic voltammogram showed these parameters as a sensitivity of 2.02 mA cm^−2^ mM^−1^, a detection limit of 58.5 μM, and a quantitation limit of 0.173 mM, with a linearity range of 0.1–1.2 mM. Table 1 compares the biosensing data for dopamine (using SWV) from the literature with the results obtained in this study.

**Table 1:** Comparative study of electrochemical biosensing metrics for dopamine of different modified electrodes

Electrode	Sensitivity (mA cm^−2^ mM^−1^)	Linearity range (mM)	Detection limit (mM)	Analyte	Ref.
EuO_3_@Cr_2_O_3_	0.0013	0.001–0.1	0.00072	DA	18
ZC31-BTC	99.5	0.0001–0.5	0.00004	DA	31
TOC/AgNPs and/or Gr	963	0.000005–0.25	0.0000005	DA	32
MoS_2_@SPE	50	0.001–0.3	0.000246	DA	33
N-Doped 4H-SiC	3.2	0.00005–0.01	0.00005	DA	34
Ni-MOF@GCE	—	0.0002–0.1	0.00006	DA	35
CeO_2_-CNTs	0.0296	0.00001–0.7	—	DA	36
LaSe@GO/GCE	0.55	0.1–1.3	0.04	DA	19
NiMoO_4_@CNTF	0.027	0.006–0.060	0.0022	DA	This study

### 3.9 Interference study of NiMoO_4_@CNTF

**Fig. 12:**
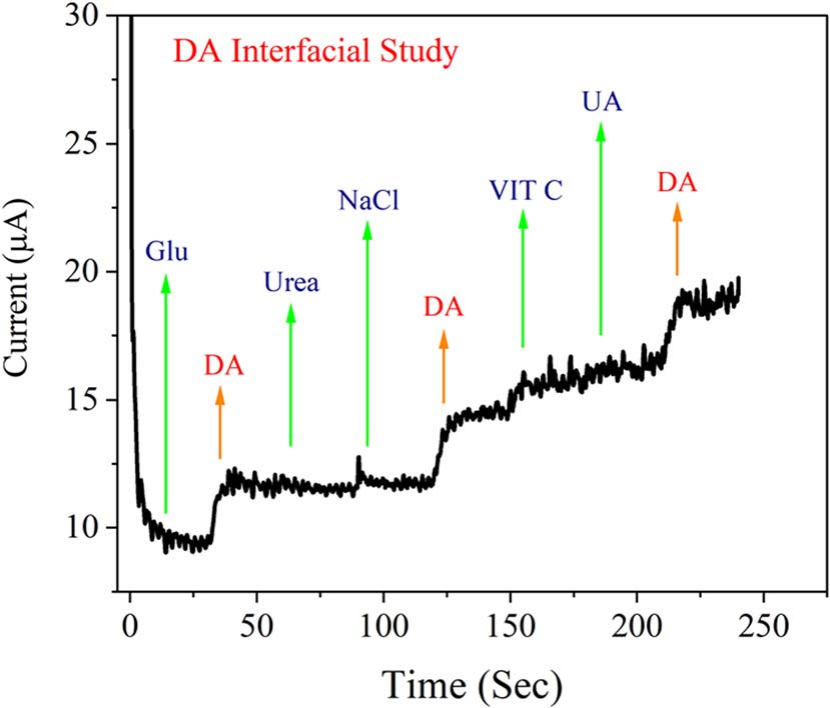
Amperometric response of the NiMoO_4_@CNTF electrode for interphase analysis of DA upon additions of Glu, Urea, UA, NaCl, AA/Vit C, and UA (20 mM).

For the analysis of real samples, the ability of the sensor to distinctly detect DA can be influenced by interfering cohabitants; *e.g.*, uric acid (UA), ascorbic acid/vitamin C (AA), sodium chloride (NaCl), glucose (Glu), and urea (CH_4_N_2_O). Therefore, in order to validate the selectivity of the NiMoO_4_@CNTF electrode towards DA, an interface analysis was performed at a specific oxidative potential of 0.12 V. Fig. 12 demonstrates the amperometric response attained by successively adding DA and other potential interferents (UA, AA, Glu, NaCl, urea). The electrode demonstrated a stable and reliable current response to DA (20 mM), and the presence of interferents with equivalent concentration did not alter the signal. In Fig. 12, a linear current response for DA can be observed, and the addition of interferents caused only negligible current variations. The obtained results validate the selectivity, reliability and robust performance of the NiMoO_4_@CNTF electrode for DA analysis, minimizing false signals in a complex sample environment.

## 4. Conclusion

A reliable and efficient NiMoO_4_@CNTF electrochemical sensor with exceptional selectivity and sensitivity for DA was successfully developed. Under the given conditions, the developed NiMoO_4_@CNTF sensor exhibited a high sensitivity of 2.02 mA cm^−2^ mM^−1^ and a detection limit of 58.2 μM, while the bare CNTF electrode showed a sensitivity of 1.4 mA cm^−2^ and a detection limit of 107 μM. The linearity ranges were 0.1–1.2 mM and 0.1–0.7 mM for the NiMoO_4_@CNTF and bare CNTF electrodes, respectively. The fabrication of CNTF with α-NiMoO_4_ nano-dots significantly enhanced the sensitivity, selectivity, surface area, conductivity, and electron transfer kinetics of the electrode for DA detection. This work may pave the way for the development of electrochemical sensors based on metal molybdates/double metal oxides with carbon-based nanomaterials and their composites for the detection of electroactive biomolecules in medical diagnosis and analysis.

## Conflicts of interest

There are no conflicts to declare.

## Supplementary Material

RA-015-D5RA05187H-s001

## Data Availability

Data will be made available on request. Supplementary information is available. See DOI: https://doi.org/10.1039/d5ra05187h.

## References

[cit1] Arif N., Gul S., Sohail M., Rizwan S., Iqbal M. (2021). Synthesis and characterization of layered Nb2C MXene/ZnS nanocomposites for highly selective electrochemical sensing of dopamine. Ceram. Int..

[cit2] Umapathi S., Masud J., Coleman H., Nath M. (2020). Electrochemical sensor based on CuSe for determination of dopamine. Microchim. Acta.

[cit3] Patella B., Sortino A., Mazzara F., Aiello G., Drago G., Torino C. (2021). *et al.*, Electrochemical detection of dopamine with negligible interference from ascorbic and uric acid by means of reduced graphene oxide and metals-NPs based electrodes. Anal. Chim. Acta.

[cit4] Bucolo C., Leggio G. M., Drago F., Salomone S. (2019). Dopamine outside the brain: the eye, cardiovascular system and endocrine pancreas. Pharmacol. Ther..

[cit5] Sajid M., Baig N., Alhooshani K. (2019). Chemically modified electrodes for electrochemical detection of dopamine: challenges and opportunities. TrAC, Trends Anal. Chem..

[cit6] Thomas J., Khanam R., Vohora D. (2015). A validated HPLC-UV method and optimization of sample preparation technique for norepinephrine and serotonin in mouse brain. Pharm. Biol..

[cit7] Hareesha N., Manjunatha J. G. (2020). Fast and enhanced electrochemical sensing of dopamine at cost-effective poly(DL-phenylalanine) based graphite electrode. J. Electroanal. Chem..

[cit8] Jie Y., Wang N., Cao X., Xu Y., Li T., Zhang X. (2015). *et al.*, Self-powered triboelectric nanosensor with poly(tetrafluoroethylene) nanoparticle arrays for dopamine detection. ACS Nano.

[cit9] Liu X., Liu J. (2021). Biosensors and sensors for dopamine detection. View.

[cit10] Patella B., Russo R. R., O'Riordan A., Aiello G., Sunseri C., Inguanta R. (2021). Copper nanowire array as highly selective electrochemical sensor of nitrate ions in water. Talanta.

[cit11] Kudłak B., Wieczerzak M. (2020). Aptamer based tools for environmental and therapeutic monitoring: a review of developments, applications, future perspectives. Crit. Rev. Environ. Sci. Technol..

[cit12] Cinti S., Colozza N., Cacciotti I., Moscone D., Polomoshnov M., Sowade E. (2018). *et al.*, Electroanalysis moves towards paper-based printed electronics: carbon black nanomodified inkjet-printed sensor for ascorbic acid detection as a case study. Sens. Actuators, B.

[cit13] Anshori I., Nuraviana Rizalputri L., Rona Althof R., Sean Surjadi S., Harimurti S., Gumilar G. (2021). *et al.*, Functionalized multi-walled carbon nanotube/silver nanoparticle (f-MWCNT/AgNP) nanocomposites as non-enzymatic electrochemical biosensors for dopamine detection. Nanocomposites.

[cit14] Norizan M. N., Moklis M. H., Demon S. Z. N., Halim N. A., Samsuri A., Mohamad I. S. (2020). *et al.*, Carbon nanotubes: functionalisation and their application in chemical sensors. RSC Adv..

[cit15] George J. M., Antony A., Mathew B. (2018). Metal oxide nanoparticles in electrochemical sensing and biosensing: a review. Microchim. Acta.

[cit16] Yang C., Denno M. E., Pyakurel P., Venton B. J. (2015). Recent trends in carbon nanomaterial-based electrochemical sensors for biomolecules: a review. Anal. Chim. Acta.

[cit17] You Y., Zou J., Li W.-J., Chen J., Jiang X.-Y., Yu J.-G. (2022). Novel lanthanum vanadate-based nanocomposite for simultaneously electrochemical detection of dopamine and uric acid in fetal bovine serum. Int. J. Biol. Macromol..

[cit18] Mijajlović A., Ognjanović M., Manojlović D., Vlahović F., Đurđić S., Stanković V. (2023). *et al.*, Eu_2_O_3_@Cr_2_O_3_ Nanoparticles-Modified Carbon Paste Electrode for Efficient Electrochemical Sensing of Neurotransmitters Precursor L-DOPA. Biosensors.

[cit19] Ahmad N., Ali A., Ahmed A. Y., Javed R., Nazir A., Akyürekli S. (2025). *et al.*, In situ growth of lanthanum selenides over graphene oxide sheets as electrocatalyst towards non-enzymatic dual detection of dopamine and ascorbic acid. J. Rare Earths.

[cit20] Zhang Y., Luo L., Zhang Z., Ding Y., Liu S., Deng D. (2014). *et al.*, Synthesis of MnCo_2_O_4_ nanofibers by electrospinning and calcination: application for a highly sensitive non-enzymatic glucose sensor. J. Mater. Chem. B.

[cit21] Liao S.-H., Lu S.-Y., Bao S.-J., Yu Y.-N., Wang M.-Q. (2016). NiMoO_4_ nanofibres designed by electrospining technique for glucose electrocatalytic oxidation. Anal. Chim. Acta.

[cit22] Chen J., Liu B., Gao X., Xu D. (2018). A review of the interfacial characteristics of polymer nanocomposites containing carbon nanotubes. RSC Adv..

[cit23] Karami C., Taher M. A. (2019). A novel enzyme-less amperometric sensor for hydrogen peroxide based on nickel molybdate nanoparticles. J. Electroanal. Chem..

[cit24] Farahpour M., Arvand M. (2021). Single-pot hydrothermal synthesis of copper molybdate nanosheet arrays as electrode materials for high areal-capacitance supercapacitor. J. Energy Storage.

[cit25] da Silva M. V., de Oliveira D. F. M., Oliveira H. S., Siqueira K. P. F. (2020). Influence of temperature on the structural and color properties of nickel molybdates. Mater. Res. Bull..

[cit26] Masteri-Farahani M., Mahdavi S., Rafizadeh M. (2013). Microemulsion-mediated synthesis and characterization of monodispersed nickel molybdate nanocrystals. Ceram. Int..

[cit27] Rammal M. B., Omanovic S. (2022). Part I: NiMoO_4_ Nanostructures Synthesized by the Solution Combustion Method: A Parametric Study on the Influence of Synthesis Parameters on the Materials’ Physicochemical, Structural, and Morphological Properties. Molecules.

[cit28] Yedluri A. K., Anitha T., Kim H.-J. (2019). Fabrication of Hierarchical NiMoO_4_/NiMoO_4_ Nanoflowers on Highly Conductive Flexible Nickel Foam Substrate as a Capacitive Electrode Material for Supercapacitors with Enhanced Electrochemical Performance. Energies.

[cit29] Ladjahirin N. A., Parimon N., Mamat M. H., Malek M. F., Uda M. N. A. (2024). Undoped and Li-Doped NiO Coral Reef-like Structures Fabricated using Immersion Method. MATEC Web Conf..

[cit30] Saberyan K., Soofivand F., Kianpour G., Salavati-Niasari M., Bagheri S. (2016). Synthesis and characterization of NiMoO_4_ via ultrasonic route by a novel precursor. J. Mater. Sci.: Mater. Electron..

[cit31] Chowdhury S., Nugraha A. S., O'May R., Wang X., Cheng P., Xin R. (2024). *et al.*, Bimetallic metal-organic framework-derived porous one-dimensional carbon materials for electrochemical sensing of dopamine. Chem. Eng. J..

[cit32] Al Kiey S. A., Khalil A. M., Kamel S. (2023). Insight into TEMPO-oxidized cellulose-based composites as electrochemical sensors for dopamine assessment. Int. J. Biol. Macromol..

[cit33] Pavličková M., Lorencová L., Hatala M., Kováč M., Tkáč J., Gemeiner P. (2022). Facile fabrication of screen-printed MoS_2_ electrodes for electrochemical sensing of dopamine. Sci. Rep..

[cit34] Fathi F., Sueoka B., Zhao F., Zeng X. (2023). Nitrogen-Doped 4H Silicon Carbide Single-Crystal Electrode for Selective
Electrochemical Sensing of Dopamine. Anal. Chem..

[cit35] Huang Z., Zhang L., Cao P., Wang N., Lin M. (2021). Electrochemical sensing of dopamine using a Ni-based metal-organic framework modified electrode. Ionics.

[cit36] Iranmanesh T., Foroughi M. M., Jahani S., Shahidi Zandi M., Hassani Nadiki H. (2020). Green and facile microwave solvent-free synthesis of CeO_2_ nanoparticle-decorated CNTs as a quadruplet electrochemical platform for ultrasensitive and simultaneous detection of ascorbic acid, dopamine, uric acid and acetaminophen. Talanta.

