# Supplementary material for: Selective non-enzymatic electrochemical detection of dopamine using nickel molybdate nano-dots anchored on CNT fiber microelectrodes
Source: RSC Adv. 2025 Oct 2;15(43):36596–606. doi: 10.1039/d5ra05187h (PMC12489750; doi:10.1039/d5ra05187h)
Supplement: RA-015-D5RA05187H-s001 [file RA-015-D5RA05187H-s001.pdf]

Supplementary Information (SI) for RSC Advances.
This journal is © The Royal Society of Chemistry 2025

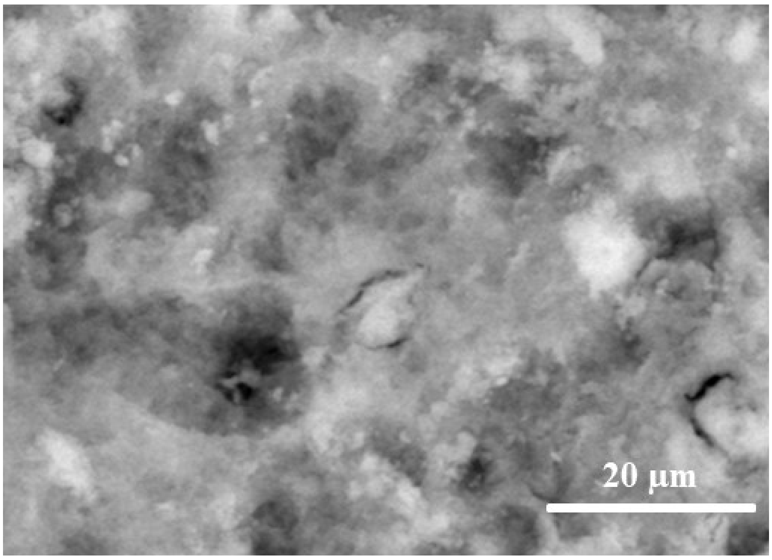

Figure S1. Post- electrochemical analysis SEM image of NiMoO4@CNTF

The post electrochemical SEM image of $NiMoO_4$@CNTF shows that nano-dot morphology is still preserved after repeated dopamine sensing measurements. Only minor surface smoothening is visible which likely arises from electrolyte interaction during cycling. The overall structural integrity confirms the mechanical robustness and electrochemical stability.
